# Case Report: A case of zolbetuximab-based chemotherapy for Claudin 18.2-positive gastric phenotype adenocarcinoma arising in Meckel’s diverticulum

**DOI:** 10.3389/fonc.2025.1700562

**Published:** 2025-11-03

**Authors:** Kohei Nagata, Takayuki Ando, Ryusuke Hanafusa, Iori Motoo, Yuko Ueda, Shinya Kajiura, Yusuke Takashima, Kenji Watanabe, Kohji Takagi, Kenichi Hirabayashi, Ichiro Yasuda

**Affiliations:** ^1^ Third Department of Internal Medicine, University of Toyama, Toyama, Japan; ^2^ Inflammatory Bowel Disease Department of Internal Medicine, University of Toyama, Toyama, Japan; ^3^ Department of Pathological Diagnosis, University of Toyama, Toyama, Japan

**Keywords:** zolbetuximab, Meckel’s diverticulum, gastric cancer, case report, Claudin18.2 (CLDN18.2)

## Abstract

A 74-year-old man presented with abdominal bloating and was diagnosed with unresectable advanced adenocarcinoma from a Meckel’s diverticulum with distant lung metastases and peritoneal dissemination. On immunohistochemical analysis, the tumor was positive for MUC5AC and MUC6 and negative for MUC2 and CDX2, indicating a gastric phenotype. Histological examination of the adjacent non-cancerous tissues confirmed the presence of ectopic gastric epithelium, resulting in a final diagnosis of adenocarcinoma arising from ectopic gastric tissue within a Meckel’s diverticulum. Biomarker profiling demonstrated Claudin 18.2 (CLDN18.2) positivity, HER2 negativity, and mismatch repair proficiency. Treatment was started with zolbetuximab plus 5-fluorouracil/leucovorin/oxaliplatin (FOLFOX), which is typically used for CLDN18.2-positive gastric cancer, resulting in a partial response. This is the first case of zolbetuximab-based therapy applied to adenocarcinoma from a Meckel’s diverticulum with a gastric phenotype due to a biomarker profile of gastric cancer. This case highlights the importance of identifying both the biomarker profile and tissue of origin of the tumor.

## Introduction

A Meckel’s diverticulum is the most common congenital anomaly of the gastrointestinal tract, resulting from incomplete obliteration of the vitelline duct (1). It is predominantly lined by ileal mucosa, although it may also contain ectopic tissue, such as gastric mucosa (1). Cancers arising in a Meckel’s diverticulum (2) are extremely rare, and the most common histological type is neuroendocrine tumors (NETs), followed by leiomyosarcomas, adenocarcinomas, and gastrointestinal stromal tumors (GISTs) (3). Among these, adenocarcinomas have significantly poorer overall survival (OS) compared to NETs and GISTs (3). Most of the previously published reports have focused on surgical cases (2), but very few reports have described chemotherapy for advanced, unresectable diseases. This case report describes a patient with a rare ectopic gastric-type adenocarcinoma arising in a Meckel’s diverticulum who was treated with zolbetuximab plus 5-fluorouracil/leucovorin/oxaliplatin (FOLFOX), a regimen based on therapeutic strategies for gastric cancer.

## Case report

**Figure 1:**
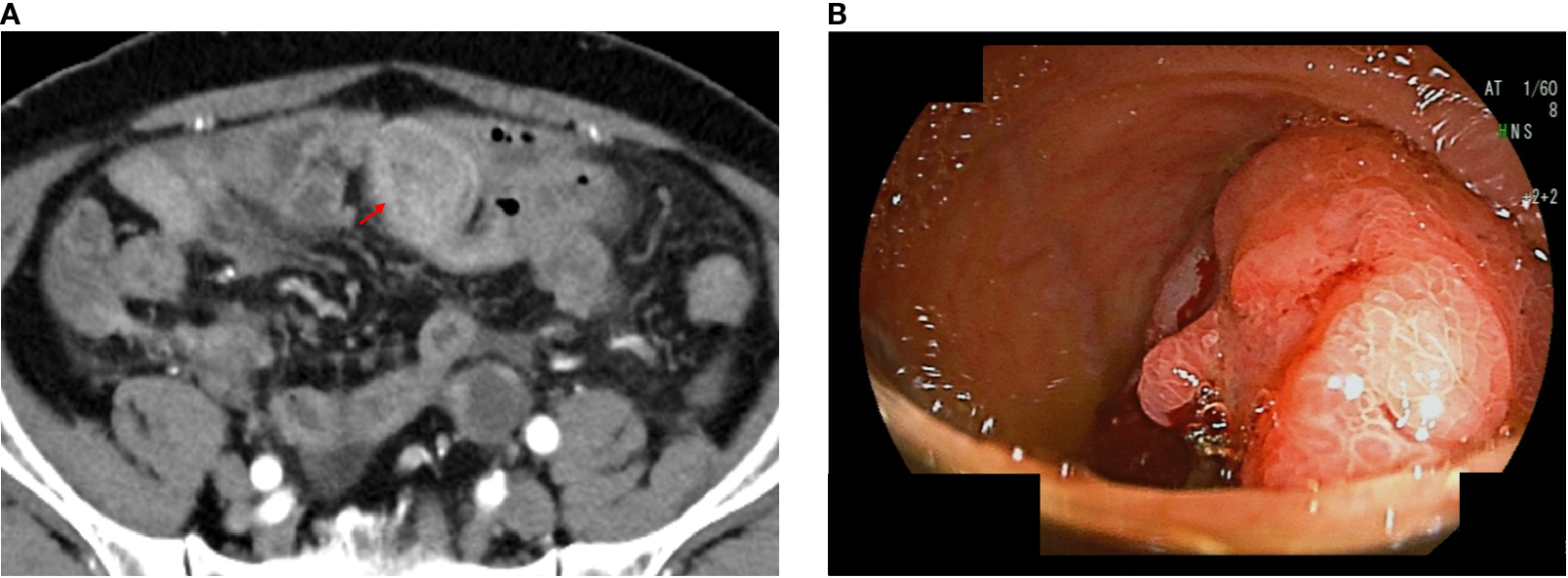
Computed tomography (CT) and endoscopic images of unresectable Meckel’s diverticulum cancer. CT images performed at our hospital; the arrow indicates an enhanced mass lesion in the ileum **(a)**. Double-balloon endoscopic finding of Meckel’s diverticulum cancer **(b)**.

A 74-year-old man presented at another hospital for abdominal bloating. His past medical history included bladder cancer, for which he underwent transurethral resection of bladder cancer at age 69, with a final pathological stage of pStage 0a (TaN0M0 according to the TNM Classification of Malignant Tumours, 9th edition), low grade. The laboratory tests were essentially normal, except for an elevated serum carcinoembryonic antigen (22 ng/mL) and carbohydrate antigen 19-9 (2,073 ng/mL). Contrast-enhanced computed tomography (CT) revealed multiple pulmonary nodules, peritoneal dissemination, mesenteric lymphadenopathy, ascites, and an enhanced mass lesion in the ileum with a 35-mm diameter (**Figure 1**). The patient was subsequently referred to our hospital for further evaluation.

**Figure 2:**
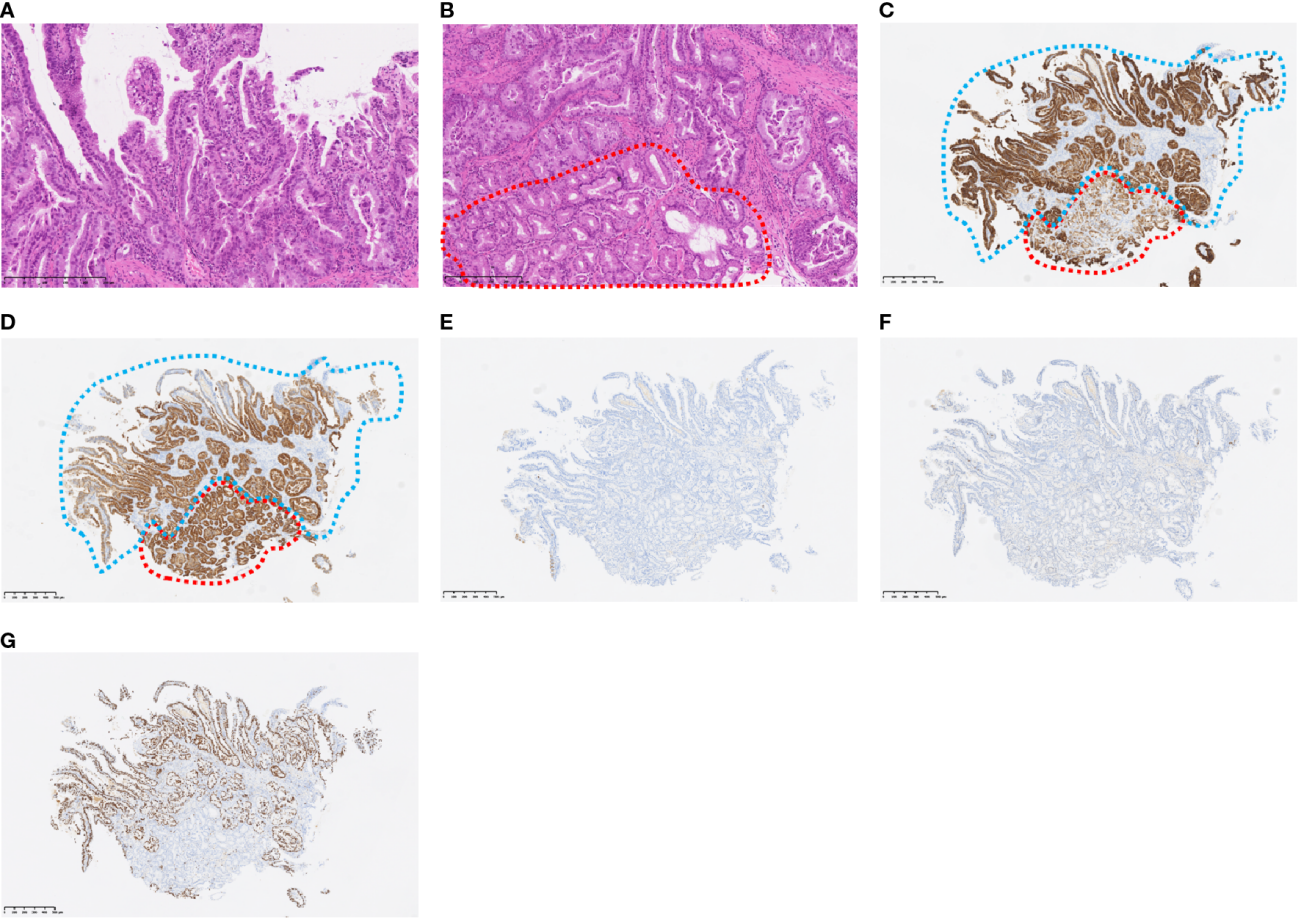
Histopathological findings at the tumor sites. The histological type of the tumor is well-differentiated carcinoma (H&E staining) **(a)** Non-tumor cells (within the red line) are in contact with the tumor cells (H&E staining) **(b)** Immunohistochemical staining of MUC5AC **(c)**, MUC6 **(d)**, MUC2 **(e)**, and CDX2 **(f)** Non-tumor cells (red dotted line) were positive for MUC5AC and MUC6, but negative for MUC2 and CDX2, indicating the presence of gastric epithelium. Tumor cells (blue dotted line) were also positive for MUC5AC and MUC6 and negative for MUC2 and CDX2, indicating gastric phenotype. Immunohistochemical staining of Ki-67, revealing 70% Ki-67-positive tumor cells, suggestive of a highly proliferative cancer type **(g)**.

Transanal double-balloon endoscopy revealed an irregular ulcer at the top with a marginal swelling (**Figure 1**), and its histological examination revealed a well-differentiated adenocarcinoma adjacent to areas of ectopic gastric epithelium (**Figure 2**). Immunohistochemistry of the tumor cells revealed positivity for MUC5AC and MUC6 and negativity for MUC2 and CDX2 (**Figure 2**). Esophagogastroduodenoscopy did not reveal any primary gastric cancer. A retrospective review of previous CT images revealed the presence of a Meckel’s diverticulum. The tumor was therefore concluded to have originated from ectopic gastric epithelium within a Meckel’s diverticulum.

**Figure 3:**
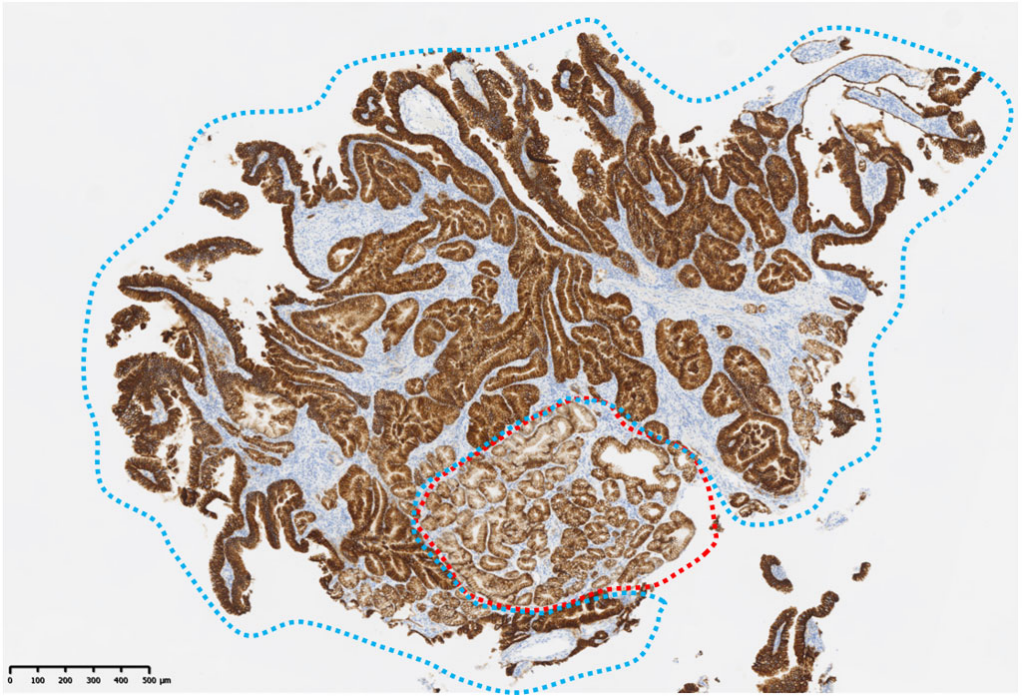
Immunohistochemical staining findings related to gastric cancer. Both non-tumor cells (red dotted line) and tumor cells (blue dotted line) are Claudin 18-positive.

**Figure 4:**
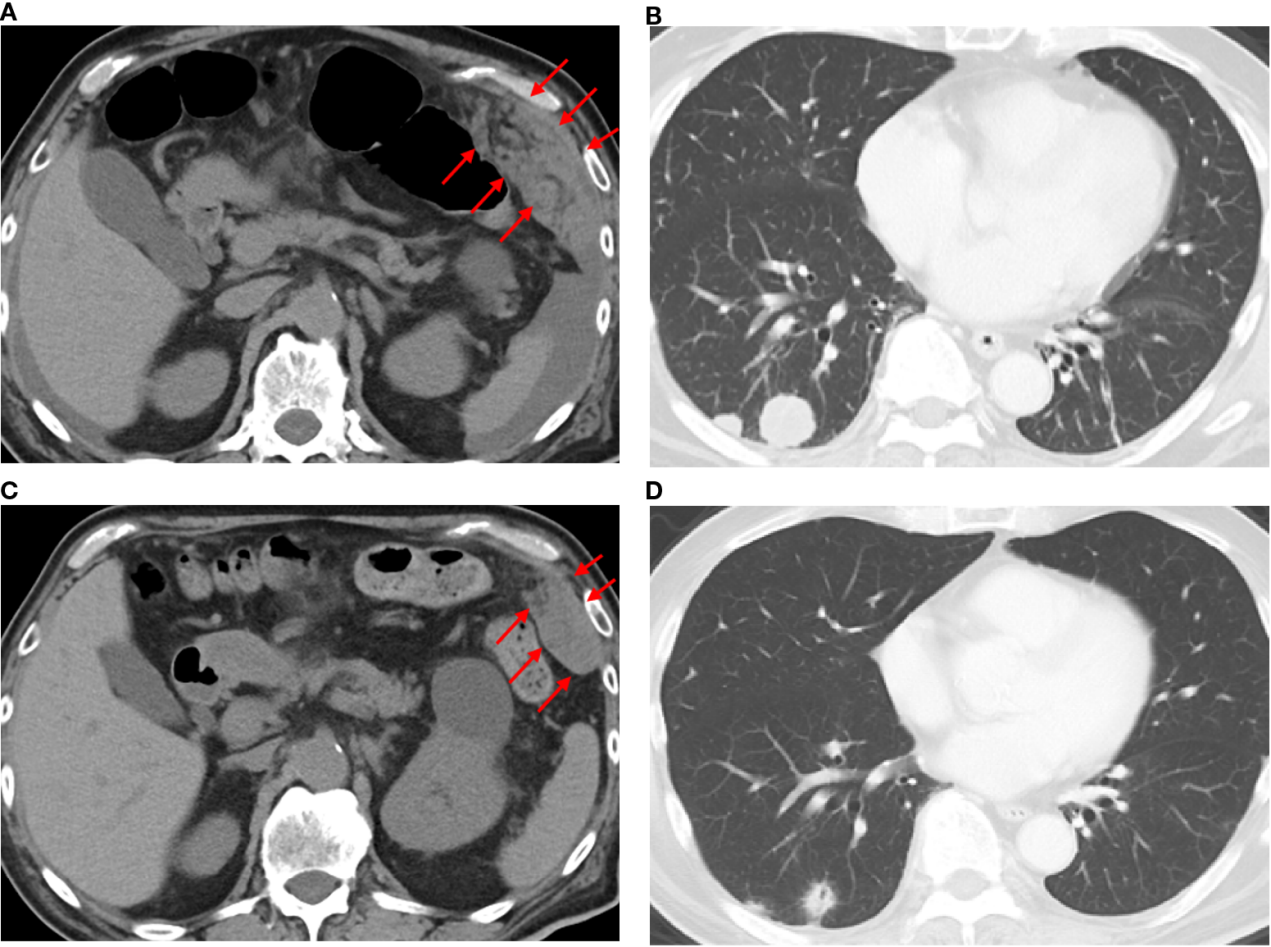
Computed tomography findings before and after the administration of zolbetuximab-based chemotherapy. CT images before treatment **(a, b)** and 2 months after zolbetuximab-based chemotherapy **(c, d)**. Red arrows indicate peritoneal disseminated nodules.

The final diagnosis was an adenocarcinoma arising in a Meckel’s diverticulum, classified as clinical Stage IV (pT3N2M1) based on the Union for International Cancer Control TNM (UICC TNM) Classification, 8th edition. This cancer diagnosis was discussed with a multidisciplinary cancer board, including a surgeon, an oncologist, and a gastroenterologist. Finally, distant metastasis was identified; therefore, chemotherapy was determined to be the optimal treatment. Additional immunohistochemical evaluations relevant to gastric cancer revealed that the tumor cells were Claudin 18.2 (CLDN18.2)-positive (**Figure 3**), HER2-negative, and mismatch repair (MMR)-proficient. Repeat CT at our institution revealed rapid tumor growth over a short period, with a Ki-67 labeling index of approximately 70% (**Figures 2**, **3**), indicating high proliferative activity. Given the gastric-type histology and CLDN18.2 positivity, combination chemotherapy with zolbetuximab plus FOLFOX was started. After 2 months of treatment, a follow-up CT scan showed a marked reduction in the size of peritoneal dissemination and pulmonary metastasis, achieving a partial response based on the Response Evaluation Criteria in Solid Tumors (RECIST) criteria (**Figure 4**). Presently, the patient is 4 months into chemotherapy with a maintained response.

## Discussion

Tumors occurring within a Meckel’s diverticulum are rare, with an estimated incidence of 3% to 6%. Among these, NETs are the most common (77.3%), followed by leiomyosarcoma (18%–25%), adenocarcinoma (12%–16%), and GIST (12%) (3). Several case reports have described an unresectable advanced Meckel’s diverticulum cancer arising from ectopic gastric epithelium (4–6). The median OS for Meckel’s diverticulum cancer is 173 months overall, and for specific subtypes, it is 243 months for NETs, 13 months for adenocarcinoma, and 62 months for leiomyosarcoma or GIST (3). Notably, adenocarcinoma has an extremely poor prognosis. Additionally, the median OS for unresectable Meckel’s diverticulum cancer is only 13 months (3), indicating even poorer outcomes for cases of unresectable adenocarcinoma. Adenocarcinomas generally exhibit more aggressive proliferation, which likely contributes to their poor prognosis. As seen in the present case, Ki-67 immunostaining revealed a high proportion of proliferative cancer cells, suggesting the aggressive nature of the tumor. There is currently no established treatment for unresectable adenocarcinoma arising within a Meckel’s diverticulum. A few reports have administered cytotoxic agents that are typically used for colorectal cancer, but these had a limited therapeutic effect (4–6). However, in recent years, chemotherapeutic strategies that target specific molecular pathways have improved outcomes in various malignancies. These suggest that biomarker-driven treatments could potentially improve the poor prognosis associated with Meckel’s diverticulum cancer.

Since Meckel’s diverticulum cancer can exhibit diverse histological subtypes, tailoring chemotherapy based on the tissue of origin of the tumor could enhance treatment efficacy (2). In particular, CLDN18.2 has recently emerged as a novel therapeutic target in gastric cancer. Two phase III trials have demonstrated that adding zolbetuximab, a monoclonal antibody targeting CLDN18.2, to first-line chemotherapy [FOLFOX or capecitabine and oxaliplatin (CAPOX)] can significantly prolong OS in patients with CLDN18.2-positive, HER2-negative gastric cancer (7, 8). In the present case, immunohistochemistry revealed that the tissues adjacent to the tumor contained normal gastric epithelial cells that were CLDN18.2-positive. Since CLDN18.2 expression is restricted to the gastric epithelium, this finding suggests the presence of ectopic gastric epithelium within the small intestine. Accordingly, zolbetuximab-based chemotherapy was selected for this case of CLDN18.2-positive adenocarcinoma with a gastric phenotype arising from ectopic gastric epithelium. A literature search was conducted in PubMed (keywords: “Meckel’s diverticulum”, “adenocarcinoma”, “chemotherapy”, and “zolbetuximab”), but no published reports have described the use of zolbetuximab-based chemotherapy for adenocarcinoma arising in a Meckel’s diverticulum.

Recently, malnutrition has been an important factor affecting the treatment response in cancer patients; therefore, nutritional care is thought to be a cornerstone of cancer treatment (9). In this case, the patient was unable to take in enough food orally due to peritoneal dissemination and ascites. However, after consulting with nutritionists, the progression of malnutrition was prevented using a combination of oral nutrition and total parenteral nutrition.

## Conclusions

This report describes our experience with zolbetuximab-based chemotherapy for Claudin 18.2-positive adenocarcinoma with a gastric phenotype arising in a Meckel’s diverticulum. This case highlights the importance of identifying the biomarker profile and tissue of origin of the tumor to optimize therapeutic efficacy, even in rare malignancies such as Meckel’s diverticulum cancer.

## Data Availability

The raw data supporting the conclusions of this article will be made available by the authors, without undue reservation.
